# IGFBP1: A Key Regulatory Gene in the Oncogenesis and Progression of Esophageal Cancer

**DOI:** 10.3390/genes17060668

**Published:** 2026-06-07

**Authors:** Jiaxin Zuo, Minmin Wen, Jiawen Li, Tao Lv, Yili Xuan, Xiwen Lu, Rongguang Zhang

**Affiliations:** 1 Key Laboratory of Tropical Translational Medicine of Ministry of Education, School of Public Health, Hainan Medical University, Haikou 571199, China; zuojiaxin0214@163.com (J.Z.); m15161678110@163.com (J.L.); 19188371060@163.com (X.L.); 2 Heinz Mehlhorn Academician Workstation, Hainan Medical University, Haikou 571199, China

**Keywords:** esophageal cancer, differentially expressed genes, IGFBP1, TCGA, GEO, prognostic value, bioinformatics analysis, cell migration

## Abstract

**Background**: Esophageal squamous cell carcinoma (ESCA) represents one of the most common aggressive malignancies worldwide. Insulin-like growth factor binding protein 1 (IGFBP1), a typical member of the IGF superfamily, is closely linked to adverse prognosis in numerous cancers. Up to now, little is known about its functional relevance to cell migration and tumor progression in ESCA. This work focuses on clarifying the relationship between IGFBP1 expression and the progression and migratory characteristics of ESCA. **Methods**: mRNA expression profiles from ESCA patients were obtained from the TCGA and GEO databases. Differential expression analysis was performed using R software(version 4.2.2), followed by an intersection of DEGs between datasets. The STRING database was applied to establish PPI networks. Cytoscape software(Version 3.7.2) was then used for visual presentation and hub gene identification. IGFBP1 expression was validated in ESCA tissues versus adjacent normal tissues. Prognostic correlation was assessed using GEPIA, while diagnostic and predictive values were evaluated through ROC analysis and Cox regression. Genetic alterations of IGFBP1 were analyzed via cBioPortal. Immune cell infiltration patterns were investigated using TIMER. Functional enrichment analyses (GO, KEGG) were performed on IGFBP1-associated DEGs. In the in vitro experiments, esophageal cancer cell lines (such as Eca109 and TE-1) and normal human esophageal epithelial cell lines (such as HEEC) were selected. The transcriptional level of IGFBP1 was examined using RT-qPCR, while Western blot analysis was conducted to validate its protein expression changes. Changes in the proliferative capacity of cancer cells after IGFBP1 silencing were detected by the CCK-8 assay, and cell migration capacity was determined via wound scratch assays to clarify the related biological effects. **Results**: Overall, 2870 DEGs were screened from the GEO database, 153 DEGs were screened from the TCGA database, and 34 genes were found to be common to both databases; 10 core genes were screened from the PPI network. IGFBP1 was abnormally expressed in esophageal cancer. Cox regression confirmed that IGFBP1 is an independent risk factor, and prognostic analysis indicated that IGFBP1 is closely associated with poor prognosis. Gene mutation analysis showed that amplification mutations are the most common type of IGFBP1 gene mutation, and genetic alterations in IGFBP1 in ESCA patients are significantly associated with overall survival (OS) (*p* = 0.0002568). GO analysis indicated that IGFBP1-related differentially expressed genes were enriched in organic anion transport, epidermal development, apical cell components, and metal ion transmembrane transporter activity. Pathway enrichment based on the KEGG database illustrated the main enrichment of target genes in neuroactive ligand–receptor interactions, calcium signaling and cAMP signaling pathways. Additionally, remarkable differences in immune cell infiltration were observed between IGFBP1 high-expression and low-expression subgroups through tumor immune profiling. IGFBP1 expression differed significantly between esophageal cancer cells and normal esophageal epithelial cells, as detected by RT-qPCR (*p* < 0.05). Moreover, knockdown of IGFBP1 markedly inhibited the proliferation (*p* < 0.05) and migration abilities (*p* < 0.05) of TE-1 and Eca109 cells. Conversely, IGFBP1 overexpression facilitated these cellular processes. Conclusions: As a key oncogenic driver for ESCA, IGFBP1 may participate in the oncogenesis of ESCA, possibly influencing clinical outcomes via IGF signaling and the tumor microenvironment. Its dual functions in tumor and immune systems suggest it might be a candidate for ESCA immunotherapy research.

## 1. Introduction

Globally, esophageal cancer (ESCA) is the eighth most common malignant tumor [1] and the sixth primary cause of cancer deaths [2]. Despite considerable progress in diagnostic techniques and therapeutic interventions for esophageal cancer (ESCA), the global five-year survival rate remains alarmingly low, ranging from 15% to 20% [3]. This persistently poor prognosis highlights ESCA as a formidable global health challenge, with its high incidence and mortality rates necessitating urgent multidisciplinary intervention strategies. From a histopathological perspective, esophageal cancer (ESCA) is primarily classified into two distinct subtypes: adenocarcinoma (AC) and squamous cell carcinoma (SCC). Notably, esophageal squamous cell carcinoma (ESCC) represents the predominant histological variant, accounting for approximately 90% of all ESCA cases globally [4]. These two histologic subtypes demonstrate fundamentally distinct (i) tumor biological behaviors, (ii) molecular pathogenesis mechanisms, (iii) clinicopathological presentations, (iv) therapeutic algorithms, and (v) epidemiological distributions. The present study systematically investigates the molecular pathogenesis of ESCA to facilitate precision diagnostics and advance targeted therapeutic development.

Insulin-like growth factor-binding protein 1 (IGFBP-1) is a member of the highly conserved IGFBP family, which comprises six homologous proteins (IGFBP-1 through IGFBP-6) [5]. The IGFBP family modulates cellular growth, survival, and proliferation through two distinct mechanisms: (1) IGF-dependent regulation via sequestration of insulin-like growth factors and (2) IGF-independent signaling through direct interaction between its conserved C-terminal arginine–glycine–aspartate (RGD) motif and cellular integrin receptors [6]. The liver serves as the primary site of IGFBP1 biosynthesis [7], but it is also expressed at low levels in the kidneys. IGFBP1 regulates cell metabolism, growth, proliferation, and migration [8] through IGF-dependent and IGF-independent mechanisms. The occurrence and development of various tumors are associated with abnormal expression of IGFBP1, such as endometrial cancer [9], thyroid cancer [10], and gastric cancer [11]. It plays a role in regulating tumor cell migration and distant metastasis and may also have a certain influence on the formation of the tumor microenvironment. IGFBP1 significantly regulates tumor cell migration and metastatic dissemination while also modulating tumor microenvironment formation. However, these mechanisms require further experimental validation and clinical confirmation.

## 2. Methods

### 2.1. Bioinformatics Analysis

#### 2.1.1. GEO and TCGA Data

We obtained 173 esophageal carcinoma (ESCA) tissue samples and 13 matched normal tissue samples from The Cancer Genome Atlas (TCGA) database through the UCSC Xena platform (https://xena.ucsc.edu/, accessed on 1 September 2025). The gene expression dataset GSE45670 was obtained from the Gene Expression Omnibus (GEO) database (https://www.ncbi.nlm.nih.gov/geo/, accessed on 2 September 2025). This dataset comprises 28 pretreatment biopsy samples from treatment-naïve ESCC patients who subsequently underwent neoadjuvant chemoradiotherapy (CRT) followed by esophagectomy and 10 matched normal esophageal mucosa samples from healthy controls. For this experiment, esophageal cancer tissue samples and normal esophageal tissue samples from the dataset were selected as samples for subsequent analysis.

#### 2.1.2. Identification of DEGs

Using the Limma package and ggplot2 package in R software (version 4.2.2), we analyzed the RNA-seq data from ESCA patients included in the TCGA database. We retrieved datasets from the GEO database and analyzed gene expression differences between normal and tumor tissues via GEO2R. All analytical results were visualized using R. DEGs were filtered according to the criteria: adjusted *p* < 0.05, FDR < 0.05 and |log_2_FC| > 1. Intersecting DEGs were selected and submitted to STRING for PPI network construction. Cytoscape was further utilized to visualize modular gene interactions and screen hub genes.

#### 2.1.3. PPI Network Construction and Visualization

A protein–protein interaction (PPI) network was constructed based on the STRING database, with a minimum required interaction score of 0.15. The resulting PPI network was then loaded into Cytoscape (Version 3.7.2) for visualization of gene–gene interactions. Within the ESCA-specific PPI network, we identified the top 10 hub genes with the highest interaction strengths (based on combined scores by the MCC method from STRING) using Cytoscape’s network analysis tools. We screened out the gene with the maximum MCC score.

#### 2.1.4. Verification of Core Gene Expression

Transcriptome profiling data and corresponding clinical information were acquired from the TCGA database, including 173 esophageal cancer (ESCA) tissue samples and 13 normal tissue samples. The differential expression of IGFBP1 was analyzed using two independent R packages, namely, limma and edgeR.

Differential gene expression analysis was further conducted using the DESeq2 package. Genes with *p* < 0.05, FDR < 0.05, and |log_2_FC| > 1 were defined as differentially expressed genes (DEGs), and the results were visualized using a volcano plot.

Based on the GEO and TCGA databases, the ggplot2 package was utilized to plot a heatmap illustrating the top 10 coexpressed genes with the strongest correlation with the target gene. The TCGA dataset served as a validation cohort to verify gene expression differences between ESCA and normal tissues.

The Shapiro–Wilk test was applied to assess the normality of the data distributions. For normally distributed data, paired Student’s *t*-tests were performed, whereas the Mann–Whitney U test was used for nonnormally distributed data. Paired-sample *t*-tests were adopted to compare expression levels of target genes between tumor tissues and paired adjacent normal tissues in ESCA patients. *p* < 0.05 was defined as statistically significant.

#### 2.1.5. Analysis of Tumor Gene Alteration Levels

Using the cBioPortal tool (https://www.cbioportal.org/, accessed on 17 September 2025), we analyzed missense mutations, truncating mutations, and gene amplifications in the TCGA-ESCA dataset. The Z-score was selected as the threshold indicator for determining genomic alterations.

#### 2.1.6. Survival Analysis

Kaplan–Meier survival curves were plotted to compare overall survival (OS) between patients with low and high IGFBP1 expression. The log-rank test was applied to evaluate survival differences, and *p* < 0.05 was defined as statistically significant. Time-dependent ROC curves were established to assess the performance of IGFBP1 in predicting 1-, 3- and 5-year survival of esophageal cancer patients.

We also utilized the GEPIA2 platform and Kaplan–Meier analysis to explore the correlation between IGFBP1 mRNA expression and overall survival.

#### 2.1.7. Analysis of Immune Cell Infiltration

To investigate the correlation between IGFBP1 expression and the immune microenvironment, the CIBERSORT algorithm was applied to analyze the proportions of tumor-infiltrating immune subpopulations in ESCA samples from the TCGA database. A profile of 21 immune cell types was constructed to characterize the immune landscape in ESCA. To further explore the correlation between IGFBP1 expression and tumor immune cell infiltration, Spearman’s correlation analysis with a 95% confidence interval (CI) was performed. The association between IGFBP1 expression and immune cell infiltration in esophageal cancer (ESCA) was validated using TIMER2.0 (http://timer.comp-genomics.org/, accessed on 29 September 2025).

#### 2.1.8. GO/KEGG Enrichment Analysis of the DEGs

Enrichment analyses of gene ontology (GO) terms and Kyoto Encyclopedia of Genes and Genomes (KEGG) pathways for differentially expressed genes, as well as functional and pathway annotation, were completed using the ClusterProfiler package (version 4.0.2).

### 2.2. In Vitro Experiments

#### 2.2.1. Cell Culture

Human normal esophageal epithelial cells (HEECs) and esophageal cancer cells (TE-1, Eca109) were maintained in RPMI 1640 medium (Procell Biotechnology, Wuhan, China) supplemented with 10% fetal bovine serum (FBS, BDBIO Biotechnology, Hangzhou, China) and 1% penicillin–streptomycin (FBS, BDBIO Biotechnology, Hangzhou, China). Cell incubation was performed at 37 °C in a humidified incubator with 5% CO_2_ to sustain regular cellular growth.

#### 2.2.2. Real-Time Quantitative Polymerase Chain Reaction (RT-qPCR) Assay

Total RNA was obtained from HEECs, TE-1, or Eca109 cells using an RNA extraction kit following the manufacturer’s instructions. Total RNA was resuspended in RNase-free water, and RNA purity and concentration were determined using a NanoDrop spectrophotometer(Thermo Fisher Scientific, Wilmington, NC, USA). One microgram (1 µg) of total RNA was reverse transcribed into complementary DNA (cDNA) with the HiScript III RT SuperMix for qPCR (+gDNA wiper) kit (Vazyme Biotech Co., Ltd., Nanjing, China). RT-qPCR was carried out on a quantitative PCR detection system using a dedicated qPCR kit. β-actin was adopted as the internal reference gene for normalization, and the relative expression levels of target genes were quantified based on the 2^−ΔΔCT^ algorithm. All experimental procedures were conducted in biological triplicates to ensure reproducibility, and the detailed primer sequences are presented in Table 1. All experiments were repeated three times. Data were presented as mean ± SD, and an independent samples *t*-test was performed using GraphPad Prism 10.0. *p* < 0.05 was considered statistically significant.

#### 2.2.3. siRNA and Plasmid Transfection

According to the endogenous expression level of IGFBP1, knockdown was performed in high-expression cell lines, and overexpression was performed in low-expression cell lines. The sequences of forward and reverse primers are listed in Table 2. Lipofectamine 3000 (Thermo Fisher Scientific, Wilmington, NC, USA)was used to transfect plasmid DNA and siRNA into the cells as instructed. For gene silencing, small interfering RNA (siRNA) targeting IGFBP1 was used to knock down endogenous IGFBP1 expression. For overexpression assays, the full-length coding sequence of IGFBP1 was inserted into the pcDNA3.1 vector between the EcoRI and NotI restriction sites. The cells were collected at 48 h after transfection for subsequent experiments.

#### 2.2.4. Western Blot

Cells were collected and lysed with ice-cold RIPA lysis buffer (Vazyme Biotech, Nanjing, China) containing 1 × protease inhibitor cocktail (Vazyme Biotech, Nanjing, China) for half an hour. Cell lysates were spun at 12,000× *g* for 10 min at 4 °C, and the supernatants containing total cellular protein were collected. Protein concentration was measured with a BCA kit. Equivalent amounts of protein samples were separated via 10% SDS-PAGE and then electrotransferred onto PVDF membranes (Bio-Rad Laboratories, Hercules, CA, USA). The membranes were blocked with 5% non-fat milk in 0.1% PBST at room temperature for 60 min, then incubated with primary antibodies targeting IGFBP1 and internal reference proteins overnight at 4 °C. After washing with PBST three times, the membranes were incubated with HRP-labeled secondary antibodies for 60 min at room temperature. Finally, protein bands were visualized by enhanced ECL chemiluminescence reagents (Vazyme Biotech, Nanjing, China) and photographed using a gel imaging system(Bio-Rad Laboratories, Hercules, CA, USA). All Western blot experiments were performed in biological triplicate to guarantee the stability and repeatability of results. The gray value of each protein band was quantitatively analyzed by image J, with β-actin serving as the internal reference. All experiments were repeated three times. Data were presented as mean ± SD, and an independent samples *t*-test was performed using GraphPad Prism 10.0. *p* < 0.05 was considered statistically significant.

#### 2.2.5. CCK-8 Assay

A CCK-8 kit (BIOSS Biotechnology, Beijing, China) was applied to evaluate cell proliferation, in line with the manufacturer’s guidelines. First, 96-well plates were seeded with 2.5 × 10^3^ cells per well. Upon completion of incubation at different time points, 110 µL fresh medium mixed with CCK-8 reagent (1:10 dilution) was added to each well. After 2 h of incubation at 37 °C in darkness, absorbance at 450 nm was measured with a microplate reader (Thermo Fisher Scientific, Wilmington, NC, USA). The experiment was repeated five times biologically. The background optical density (OD) values of the blank group were subtracted to remove interference from the culture medium and reagents. The relative cell survival rate was calculated as follows: Relative cell survival rate = (Corrected OD value of experimental group/Corrected OD value of control group) × 100%. Data were presented as mean ± standard deviation. Two-way analysis of variance (two-way ANOVA) was conducted using GraphPad Prism 10.0.

#### 2.2.6. Wound Healing Assay

Six-well plates were seeded with 3–4 × 10^4^ cells per well. When the cells grew to full confluence, a straight scratch was made with a sterile 1 mL pipette tip. The medium was changed to serum-free Opti-MEM. Scratch images were captured at 0 h and 24 h. To normalize the migration ability for accurate intergroup comparison, the relative migration rate (%) was calculated as follows: Relative migration rate (%) = (Experimental wound closure rate/Control wound closure rate) × 100%. All scratch lines and related measurements were conducted with Adobe Photoshop (PS) 2026 software. All experiments were repeated three times. Data were presented as mean ± SD and analyzed by one-way ANOVA using GraphPad Prism 10.0. *p* < 0.05 was considered statistically significant.

## 3. Results

### 3.1. COX Regression Analysis

**Figure 1:**
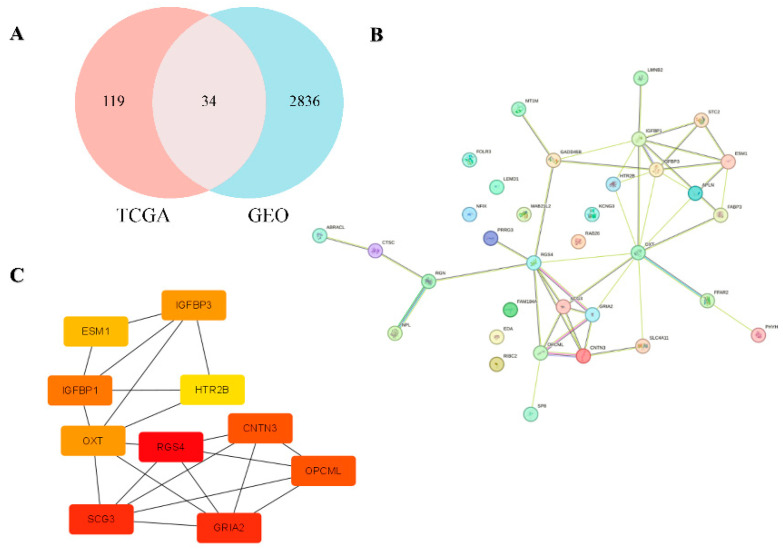
(**A**) Venn diagram showing the intersection between differentially expressed genes in the GSE45670 and TCGA databases. (**B**) Protein–protein interaction prediction map. (**C**) Screen for 10 core genes in a key position in the PPI network by the cytoHubba plugin.

A total of 2870 differentially expressed genes (DEGs) were screened from the GEO database cohort. Univariate Cox regression analysis was performed to assess their association with survival in ESCA patients, identifying 153 DEGs with significant prognostic value. Intersection analysis with the initial 2870 GEO-derived DEGs yielded 34 genes showing significant differential expression (Figure 1A).

### 3.2. Protein Interaction Network Analysis of Differentially Expressed Genes

To further explore the underlying mechanisms, a protein–protein interaction (PPI) network was constructed using the STRING tool with a minimum interaction score threshold of 0.4 (Figure 1B). Using Cytoscape software integrated with the STRING database, we identified genes exhibiting strong interactions with IGFBP1 in the PPI network: RGS4, GRIA2, SCG3, OPCML, CNTN3, IGFBP1, OXT, IGFBP3, ESM1, and HTR2B. The interaction network among these genes was visualized to delineate their functional interrelationships (Figure 1C) Notably, IGFBP1 ranked first with the maximum MCC score.

### 3.3. Differentially Expressed Core Genes

**Figure 2:**
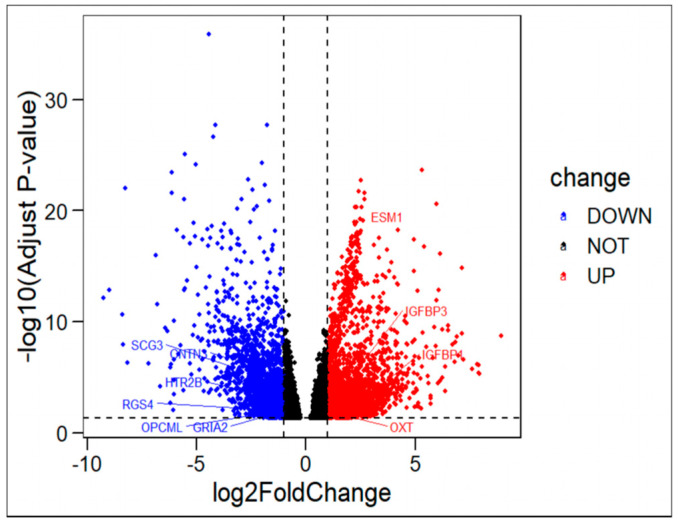
Differential gene volcano map.

**Figure 3:**
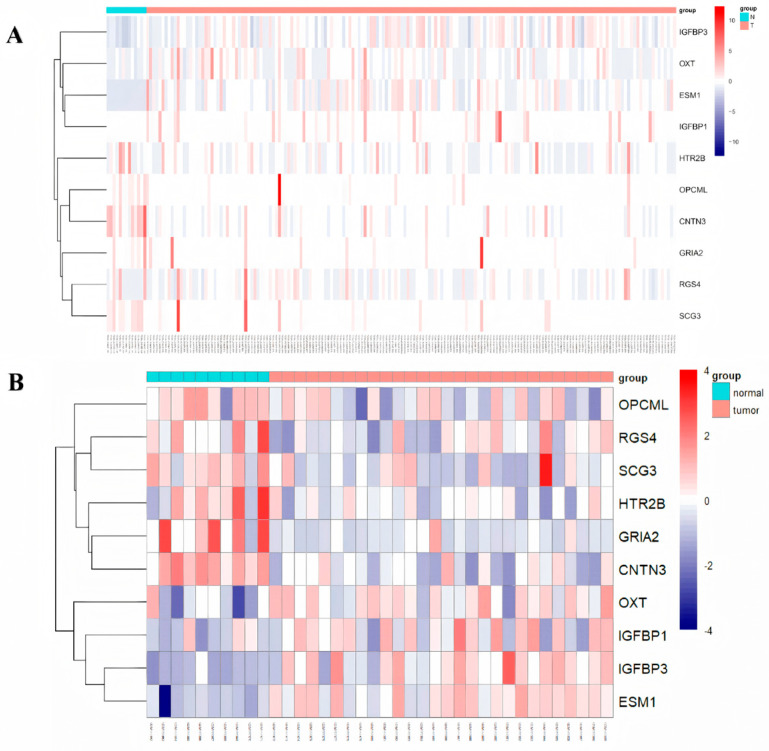
(**A**) Heatmap of significantly up- and downregulated genes in the intersecting gene TCGA. (**B**) Heatmap of significantly up- and downregulated genes in the intersecting gene GEO.

**Figure 4:**
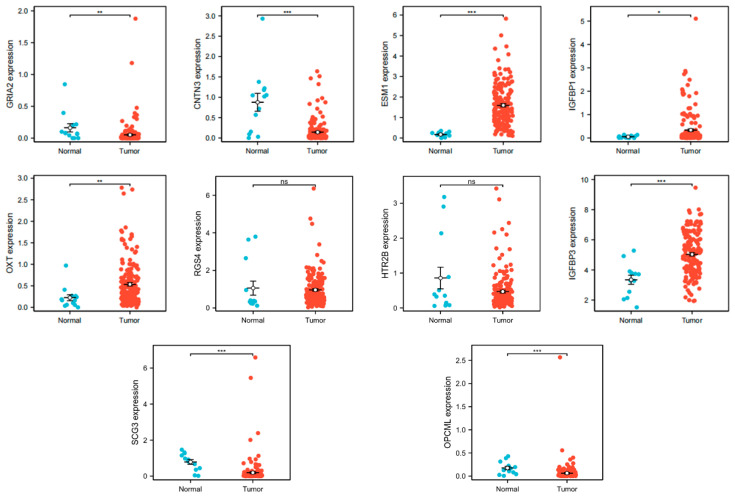
Differential expression of 10 genes in TCGA in paraneoplastic and cancerous tissues (as unpaired). *, *p* < 0.05, **, *p* < 0.01; ***, *p* < 0.001, ns: *p* > 0.05.

**Figure 5:**
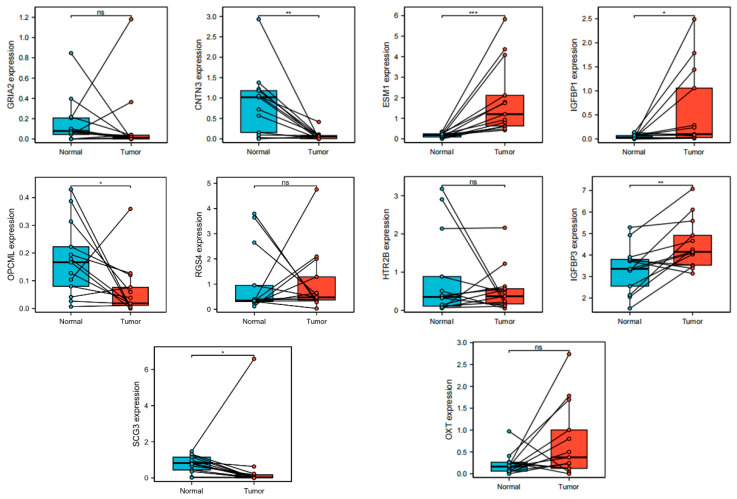
Differential expression of 10 genes in TCGA in paraneoplastic and cancerous tissues (as paired).*, *p* < 0.05; **, *p* < 0.01; ***, *p* < 0.001, ns: *p* > 0.05.

The expression levels of the 10 selected genes in the TCGA database were analyzed using the R language package. Among the 10 genes, IGFBP1, OXT, IGFBP3, and ESM1 were all highly expressed in esophageal cancer, while RGS4, GRIA2, SCG3, OPCML, CNTN3, and HTR2B were all lowly expressed in esophageal cancer, with *p* < 0.05 and |log_2_FC| > 1, indicating a statistically significant difference.The TCGA volcano plot (Figure 2) revealed that HTR2B, GRIA2, and CNTN3 were significantly downregulated in tumor samples but upregulated in normal tissues, clustering into one subgroup. Conversely, OXT, IGFBP1, IGFBP3 (validated by *T*-test), and ESM1 were significantly upregulated in tumors and downregulated in normal tissues, forming a distinct cluster. To validate these findings, we analyzed the expression levels of these ten genes in an independent cohort of 173 gastric cancer tissues and 13 adjacent normal tissues (including both paired and unpaired samples) from the TCGA database(Figure 3A), as well as a GEO dataset with 10 controls and 28 cases (Figure 3B). Among the two methods mentioned above (Figure 4), the expression levels of IGFBP1, ESM1, and IGFBP3 proteins in esophageal cancer tissues were significantly higher than those in adjacent normal tissues (Figure 5).

### 3.4. The Relationship Between IGFBP1 Gene Mutations and Prognosis in ESCA Patients

**Figure 6:**
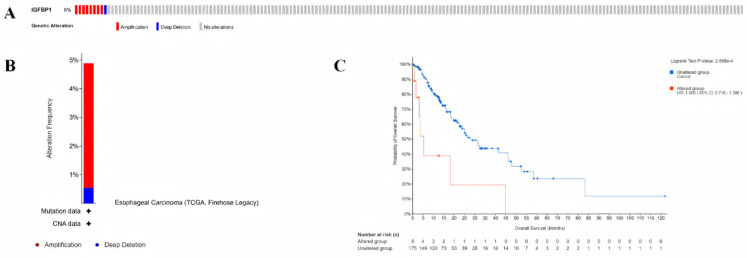
(**A**) Gene mutations of IGFBP1 in ESCA. (**B**) Frequency of gene mutations of IGFBP1 in ESCA. (**C**) Comparison of overall survival between patients with and without mutations in the IGFBP1 gene.

We leveraged the cBioPortal platform to analyze and visualize RNA-seq data from ESCA patients in the TCGA dataset, aiming to investigate the impact of IGFBP1 gene mutations on patient prognosis. In ESCA patients, IGFBP1 gene mutations were observed in nine cases (4.89%), including one case (0.54%) with a deep deletion mRNA mutation and eight cases (4.35%) with amplification mutations (Figure 6A,B). Survival analysis demonstrated that IGFBP1 gene mutations in ESCA patients were significantly associated with overall survival (Figure 6C, *p* = 2.568 × 10^−4^ < 0.05), exhibiting a positive correlation. Among mutation types, amplification mutations were the most prevalent, and ESCA patients with IGFBP1 mutations showed a significant association with worse OS outcomes.

### 3.5. Diagnostic and Prognostic Value of IGFBP1 Expression in ESCA

**Figure 7:**
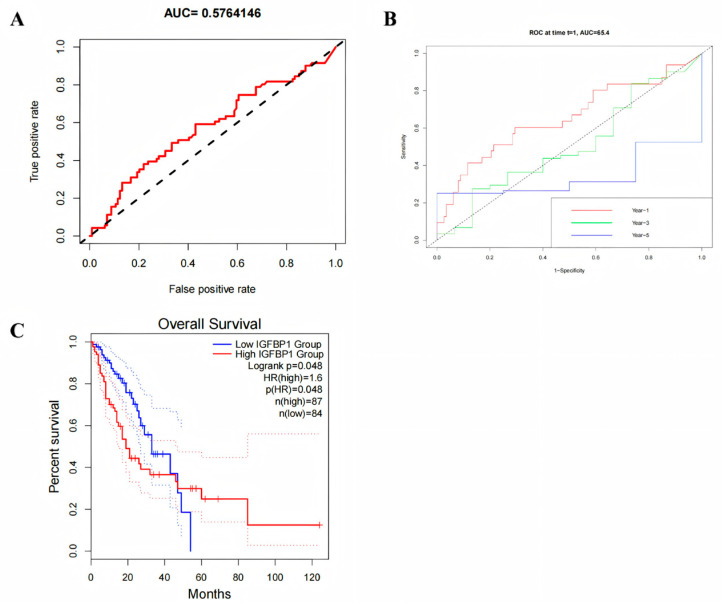
(**A**) Diagnostic ROC curve of IGFBP1 for ESCA in the TCGA dataset. (**B**) IGFBP1 model time ROC curve for esophageal cancer patients in the TCGA dataset. (**C**) TCGA database and Kaplan–Meier analysis of the survival relationship in patients with IGFBP1-expressing ESCA.

Univariate Cox regression analysis showed that IGFBP1 expression was significantly associated with overall survival (OS; hazard ratio [HR] = 1.405, 95% confidence interval [CI] = 1.043–1.893, *p* = 0.003). Further analysis of its diagnostic performance via an ROC curve revealed limited diagnostic value for ESCA (Figure 7A, AUC = 0.576). A time-dependent ROC curve was constructed to predict 1-, 3-, and 5-year survival in HCC patients, demonstrating good predictive performance for 1-year survival (Figure 7B, AUC = 0.654). Using the GEPIA2 platform, K-M survival analysis was conducted to generate a curve depicting the correlation between IGFBP1 expression and overall survival. The results showed that ESCA patients with high IGFBP1 expression had significantly poorer OS [Figure 7C, HR = 1.60 (95% CI: 1.15–2.37), *p* = 0.048 < 0.05]. These results imply that IGFBP1 dysregulation may be a potential auxiliary prognostic indicator for ESCA, rather than a robust and independent clinical biomarker.

### 3.6. Correlation Analysis Between IGFBP1 Expression Level and ESCC Immune Infiltration Abundance

**Figure 8:**
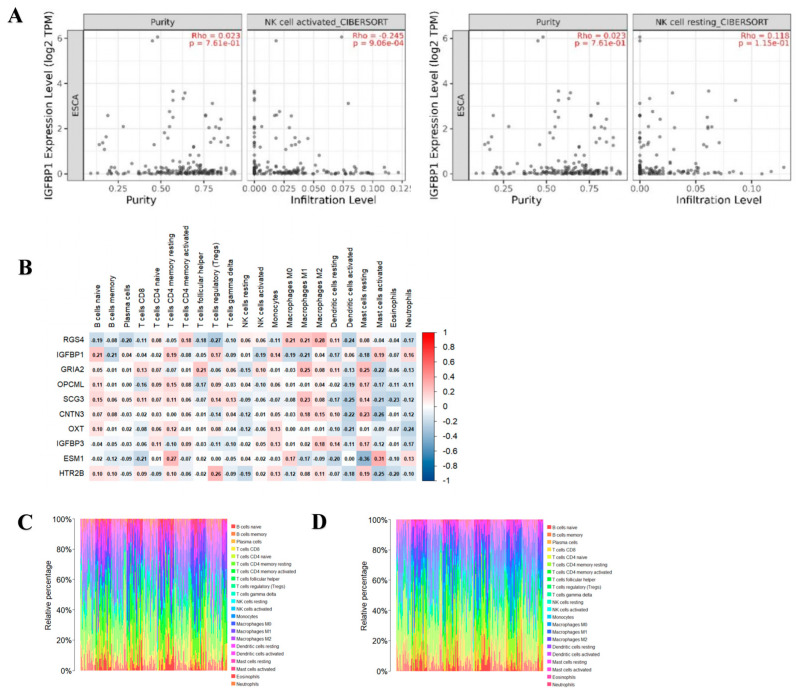
(**A**) Correlation of IGFBP1 with NK cell activation and NK cell resting infiltration. (**B**) Correlation analysis of tumor-infiltrating immune cells (TICs). (**C**) ESCA tumor-infiltrating immune cell atlas. (**D**) Normal cell-infiltrating immune cell atlas.

Compared to normal immune cell proportions (Figure 8D), esophageal cancer tissues (Figure 8C) exhibited significantly reduced frequencies of mast cells, macrophages, resting dendritic cells, and activated natural killer cells.

Among these, six types of TICs showed a positive correlation with IGFBP1 expression. These results support the conclusion that IGFBP1 levels influence immune activity in ESCA (Figure 8B).

**Figure 9:**
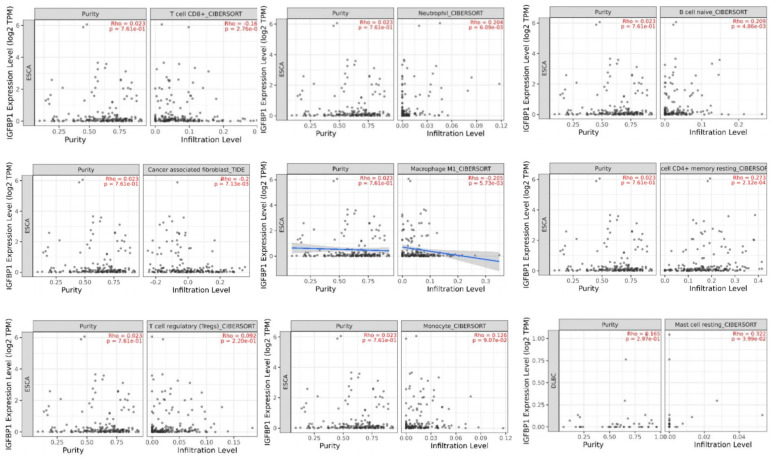
Correlation of IGFBP1 with respective immune cell infiltration.

Analysis of the TIMER database revealed that IGFBP1 expression in TCGA-ESCA was positively correlated with NK cells (Figure 8A, r = −0.245, *p* > 0.05), B lymphocytes (r = 0.209, *p* < 0.05), resting memory CD^4+^ T cells (r = 0.273, *p* < 0.05), monocytes (r = 0.126, *p* < 0.05), resting mast cells (r = 0.322, *p* < 0.05), and neutrophils (r = 0.204, *p* < 0.05) in the tumor microenvironment (TME). Conversely, negative correlations were observed with CD8^+^ T lymphocytes (r = −0.164, *p* < 0.05), M1 macrophages (r = −0.205, *p* < 0.05), and activated natural killer cells (r = −0.245, *p* < 0.05). No significant correlations were found with M0 macrophages, regulatory T cells, or resting natural killer cells (*p* > 0.05). Given the potential oncogenic role of IGFBP1 in ESCA, we further analyzed its association with cancer-associated fibroblasts (CAFs) using TIMER, revealing an inverse correlation between IGFBP1 expression and CAF abundance (Figure 9, r = −0.2, *p* < 0.05). These findings suggest that IGFBP1 is involved in regulating immune cell infiltration within the tumor immune microenvironment of ESCA. However, the specific molecular mechanisms underlying its role in ESCA tumorigenesis and progression require further investigation to be fully elucidated.

### 3.7. IGFBP1 Is Associated with Biological Processes and Pathways in Esophageal Cancer Cells

**Figure 10:**
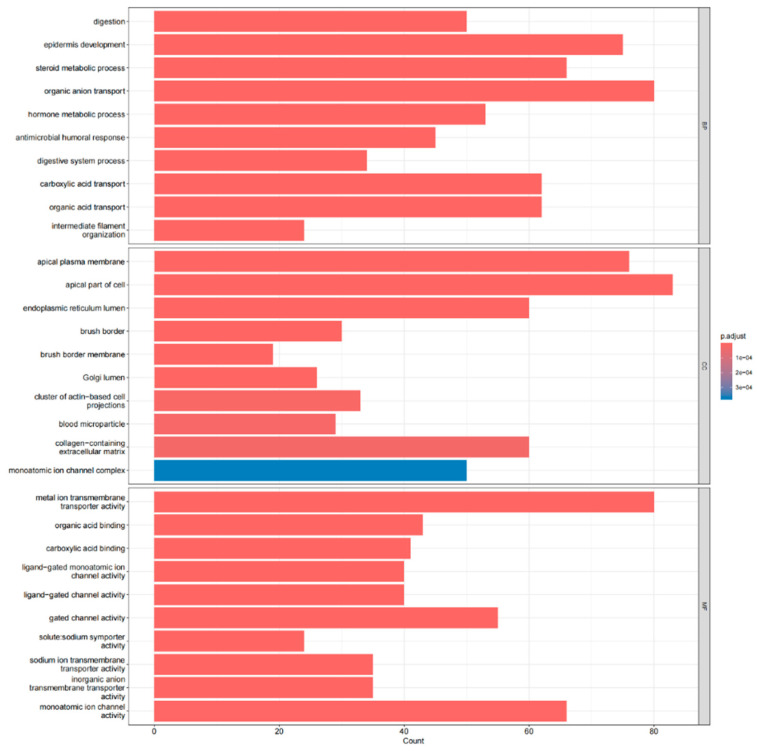
GO pathway analysis of genes associated with IGFBP1 expression in ESCA.

**Figure 11:**
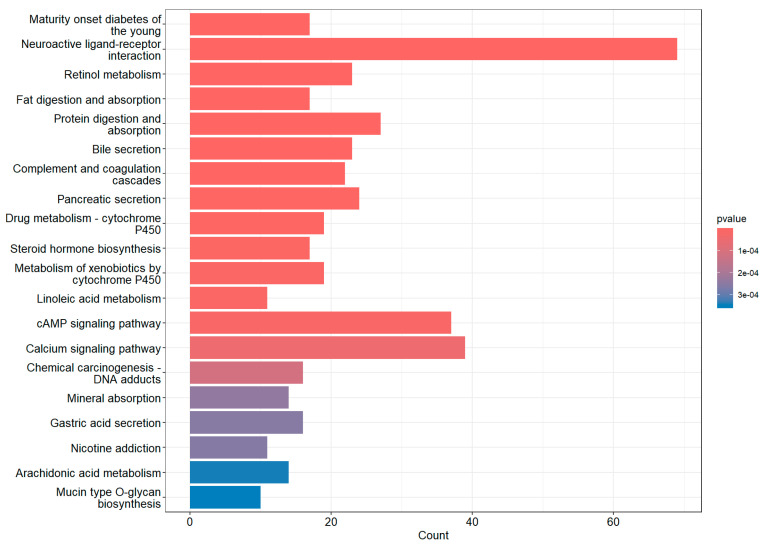
KEGG pathway analysis of genes associated with IGFBP1 expression in ESCA.

To elucidate the potential biological processes and pathways linked to IGFBP1 in ESCA, GO enrichment analysis was performed covering three major categories: Biological Process (BP), Cellular Component (CC), and Molecular Function (MF). The results revealed that in the BP category, organic anion transport, epidermis development, and steroid metabolic process were significantly enriched; in the CC category, the apical part of the cell and the apical plasma membrane were prominently enriched; and in the MF category, IGFBP1 was primarily associated with metal ion transmembrane transporter activity and monoatomic ion channel activity (Figure 10). KEGG enrichment analysis indicated that the neuroactive ligand–receptor interaction, calcium signaling pathway, and cAMP signaling pathway are potentially involved in the tumorigenic process of IGFBP1 in ESCA (Figure 11).

### 3.8. Knockdown of IGFBP1 Inhibits the Proliferation of ESCA Cells

**Figure 12:**
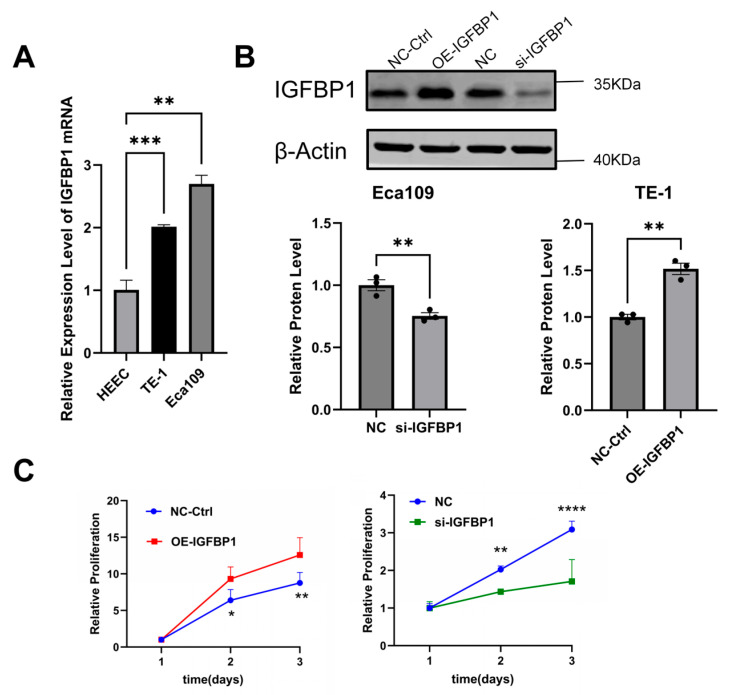
(**A**) The overexpression of IGFBP1 in TE-1 and Eca109 cells was verified by real-time quantitative polymerase chain reaction (RT-qPCR). (**B**) Western blotting was used to verify the knockdown and overexpression efficiency of IGFBP1. (**C**) CCK-8 assay results showed that IGFBP1 overexpression significantly promoted the proliferation of TE-1 cells, while IGFBP1 knockdown inhibited the proliferation of Eca109 cells. *, *p* < 0.05; **, *p* < 0.01; ***, *p* < 0.001; ****, *p* < 0.0001.

IGFBP1 was knocked down in Eca109 and TE-1 cells via siRNA transfection, and the knockdown efficiency was verified by RT-qPCR (Figure 12A) and Western blot (Figure 12B) analysis, respectively. CCK-8 assays (Figure 12C) showed that, compared with the siNC group, knockdown of IGFBP1 significantly suppressed the proliferative capacity of Eca109 and TE-1 cells. Collectively, these results indicate that silencing IGFBP1 inhibits the proliferation of ESCA cells.

#### 3.8.1. Knockdown of IGFBP1 Impairs the Migration Ability of ESCA Cells

Invasive growth is a hallmark of ESCA progression. The wound-healing assay was performed to evaluate the migratory capacity of Eca109 and TE-1 cells. The results demonstrated that knockdown of IGFBP1 significantly inhibited cell migration.

#### 3.8.2. Overexpression of IGFBP1 Promotes the Migration of ESCA Cells

**Figure 13:**
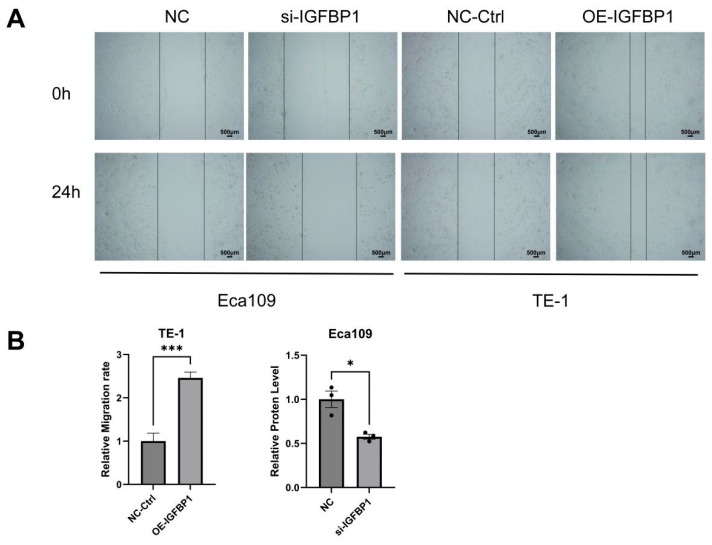
IGFBP1 regulates ESCA cell migration through overexpression and knockdown. (**A**) Wound healing assay results of ESCA cell migration following IGFBP1 overexpression. (**B**) The migratory capacity of ESCA cells was evaluated by the wound healing assay after IGFBP1 knockdown. *, *p* < 0.05; ***, *p* < 0.001.

Based on the above knockdown results, we hypothesized that IGFBP1 overexpression would enhance ESCA cell migration. IGFBP1 exhibited upregulated expression in Eca109 and TE-1 cells using the pcDNA3.1 vector, and wound healing assays were subsequently performed(Figure 13A). Compared with the empty vector control group, IGFBP1 overexpression significantly promoted cell migration(Figure 13B). Taken together, these gain- and loss-of-function experiments confirm that IGFBP1 plays a critical role in promoting the migration of ESCA cells.

## 4. Discussion

Esophageal cancer has emerged as a significant global health challenge, characterized by its high incidence rate [12], dismal prognosis [13], and a notable scarcity of targeted research and effective therapeutic strategies. Additionally, the acquisition and progression of chemotherapy resistance represent a critical barrier to improving treatment outcomes [14]. The gold standard for diagnosing esophageal cancer is pathological examination, which involves acquiring pathological specimens via esophageal endoscopy and microscopically observing cellular morphological and structural characteristics to identify the presence of cancer cells, as well as determine their type and differentiation degree [12]. This approach remains the most reliable method for confirming an esophageal cancer diagnosis.

However, endoscopy is an invasive procedure, which limits its widespread use as a screening tool for the asymptomatic population [15]. With the increasing incidence of esophageal cancer, there is an urgent need for novel approaches that enable early detection [16], effective prevention, and precise treatment [17].

In this study, gene expression profiles of esophageal cancer were collected from the TCGA and GEO databases for differential expression analysis. The analysis identified 153 and 2870 differentially expressed genes (DEGs) in esophageal cancer (ESCA) tissues from the TCGA and GEO databases, respectively, which were subjected to downstream analysis. Subsequently, 10 hub genes were identified through protein–protein interaction (PPI) network analysis, potentially providing diagnostic and prognostic insights for ESCA patients.

The available literature has documented that aberrant expression of IGFBP1 is linked to the pathogenesis of multiple tumors, including gastric cancer [18], endometrial cancer [9] and lung adenocarcinoma [19]. However, the association between IGFBP1 dysregulation and the initiation/progression of esophageal cancer remains poorly understood. This study aims to explore the correlation between elevated IGFBP1 expression and ESCA development and progression and to speculate on its potential underlying mechanisms based on published evidence.

The insulin-like growth factor (IGF) signaling pathway (IGF-IGFR-IGFBP axis) comprises six distinct insulin-like growth factor-binding proteins (IGFBPs), the classic ligands insulin-like growth factor 1 (IGF1) and insulin-like growth factor 2 (IGF2) [20], IGF receptors (IGF-IR/IGF1R and IGF-IIR/IGF2R), and multiple IGFBP proteases, such as kallikrein, cathepsin, and matrix metalloproteinases (MMPs) [21]. The IGF signaling pathway is crucial for cellular differentiation, growth, and apoptosis. IGFBPs (insulin-like growth factor-binding proteins) are cysteine-rich proteins that bind to IGFs with high affinity, modulating IGF signaling in a cell type-dependent manner—either enhancing or inhibiting pathway activity [22]. IGFBP1 is a key component of this signaling system. As the primary form of IGFBP, it can infiltrate tissues via autocrine and paracrine mechanisms. Previous studies have demonstrated that IGFBP1 regulates cell proliferation, migration, and autophagy by modulating IGF [23] and IGFR levels [24]. The IGF signaling pathway mediates cell–environment communication, contributes to human cancer progression, and represents a promising therapeutic target [25]. Accumulated studies suggest that IGFBP1 may interact with β1 integrin via its RGD-binding motif and indirectly regulate IGF bioactivity, which could potentially affect cancer cell migration. In addition, IGFBP1 can form a complex with IGF-1 to prolong its half-life and modulate its availability, which is presumed to participate in the regulation of cell proliferation and apoptosis. Based on the existing literature, IGFBP1 may modulate a variety of cellular events, including cell proliferation, apoptosis, DNA damage repair and tumorigenesis, through both IGF-dependent and IGF-independent manners. Further experimental validation is still required to confirm these observations [26]. Notably, studies in other tumor types (e.g., glioblastoma [27]) have shown that macrophage colony-stimulating factor (MCSF) secreted by glioblastoma cells enhances IGFBP1 secretion by microglia, with IGFBP1 serving as a key mediator of tumor angiogenesis. By contrast, serum IGFBP1 levels are downregulated [28] in ovarian cancer and hepatocellular carcinoma [29] but upregulated in nasopharyngeal carcinoma [30]. Studies have demonstrated that serum IGFBP1 levels are associated with cardiovascular diseases [31] and gestational diabetes [32]. These findings in nonneoplastic diseases underscore the pleiotropic roles of IGFBP1 in human pathology and highlight its potential relevance to esophageal cancer tumorigenesis. These findings reveal the diverse biological functions of IGFBP1, while more evidence is still needed to verify its exact role in esophageal tumorigenesis.

Comprehensive PPI network analysis has yielded novel insights into the protein interaction landscape and potential therapeutic targets of IGFBP1 in esophageal cancer. Notably, nine proteins were identified as closely associated with IGFBP1: RGS4, GRIA2, SCG3, OPCML, CNTN3, OXT, IGFBP3, ESM1, and HTR2B. Among these proteins, a study by Kang Kui et al. [3] revealed that ESM1 suppresses cell viability, migration, and invasion in human gastric adenocarcinoma (AGS) and esophageal cancer (TE1) cells, while upregulating apoptosis in both cell lines. Mechanistically, ESM1 silencing promotes the progression of stomach adenocarcinoma (STAD) and esophageal cancer (ESCA). Clinically, high ESM1 expression correlates with poor prognosis in STAD and ESCA patients, and pan-cancer analysis confirms its significant association with overall survival (OS) in ESCA and other malignancies.

IGFBP3 is a multifunctional carrier protein that regulates cell proliferation and apoptosis via both IGF-dependent and IGF-independent pathways. A study by Natsuizaka M et al. [33] identified IGFBP3 as a hypoxia-inducible gene that modulates diverse cellular processes, including proliferation, senescence, apoptosis, and epithelial–mesenchymal transition (EMT). Mechanistically, IGFBP3 suppresses reactive oxygen species (ROS) through an IGF-independent mechanism, fostering a hypoxic microenvironment. This process mediates the induction of CD44H+ cells, thereby promoting the progression of esophageal squamous cell carcinoma. Williams AC [34], Hollowood AD [35], and other researchers have proposed that insulin-like growth factor-binding protein-3 (IGFBP-3) may function as a tumor suppressor in human esophageal squamous cell carcinoma (ESCC). However, its role in ESCC remains controversial, as IGFBP-3 has been implicated in both pro-tumorigenic and anti-tumorigenic effects. Except for ESM1 and IGFBP3, the roles of the other identified genes in cancer progression, cell differentiation, invasion/metastasis, immune evasion, and treatment response remain unclear. Cluster analysis grouped IGFBP1, OXT, IGFBP3, and ESM1 together, implying their potential collaboration in biological pathways critical for tumorigenesis and progression. Notably, as members of the IGF system, IGFBP1 and IGFBP3 act via autocrine/paracrine mechanisms to infiltrate tissues. Concurrently, OXT and ESM1 may engage in tumor microenvironment-related pathways through similar secretory mechanisms, interacting with the IGF axis to collectively modulate tumor cell behavior—including promotion of proliferation, migration, and angiogenesis.

This study analyzed IGFBP1 expression and clinical data from the TCGA database, revealing that aberrant IGFBP1 expression is significantly associated with reduced overall survival (OS) in esophageal cancer patients (hazard ratio [HR] = 1.40513, 95% confidence interval [CI] = 1.043132–1.892753, *p* = 0.003). ROC analysis yielded an AUC of 0.5764146, indicating that the diagnostic efficacy of single IGFBP1 for ESCA is relatively limited and cannot achieve high accuracy for clinical diagnosis. Although IGFBP1 exhibited a relatively higher AUC value of 0.654 for predicting 1-year survival, its predictive power remains modest and only provides preliminary suggestive prognostic information, which is partially consistent with the findings of a previous study by Xu YW et al. [36]. Kaplan–Meier survival analysis further demonstrated that elevated IGFBP1 expression was marginally associated with poorer OS (HR = 1.6, *p* = 0.048). Collectively, these results imply that IGFBP1 dysregulation may be a potential auxiliary prognostic indicator for ESCA, rather than a robust and independent clinical biomarker.

CD8^+^T cells and activated NK cells are the core effector cells responsible for anti-tumor immunity. A reduction in their number directly impairs the body’s capacity to monitor and eliminate tumor cells. M1 macrophages exert pro-inflammatory and tumor-suppressive effects, and decreased infiltration of this cell subset further exacerbates local immune dysfunction. This study revealed that the high IGFBP1 expression group exhibited a significant increase in B lymphocytes, resting memory CD^4+^ T cells, monocytes, resting mast cells, and neutrophils, accompanied by a notable decrease in CD8^+^ T cells, M1 macrophages, and activated natural killer cells. Accumulating studies [37] have shown that IGFBP family proteins participate in signal communication between tumor cells and immune cells via secretory pathways and regulate the activation, differentiation and functional phenotypes of immune cells. Accordingly, we hypothesize that highly expressed IGFBP1 in esophageal cancer acts on various immune cells through paracrine and autocrine mechanisms, alters immune cell infiltration patterns and biological functions, and ultimately mediates tumor immune escape to facilitate tumor initiation, invasion and progression. Combining our correlation results and the previous literature, we tentatively hypothesize that highly expressed IGFBP1 in ESCA may act on various immune cells through paracrine and autocrine pathways, alter immune cell infiltration and biological functions, and potentially mediate tumor immune escape to facilitate tumor development. The above regulatory mechanisms have not been verified by experiments.

Although this study confirms the correlation between IGFBP1 expression and immune infiltration, the direct regulatory mechanism between them remains to be verified experimentally. Restricted by experimental conditions, we did not perform functional experiments such as in vitro cell co-culture and in vivo animal models, so the specific molecular targets and signaling pathways by which IGFBP1 regulates immune cells have not been clarified. Further research will focus on exploring the mechanism of IGFBP1 in the tumor immune microenvironment and provide novel theoretical evidence for the development of IGFBP1-targeted tumor immunotherapy.

Tumor cell proliferation disorders triggered by key gene mutations often drive tumorigenesis. Studies by El Tekle G [38] and Rogier M [39] have shown that FAM72A overexpression may skew the balance toward mutagenic DNA repair, rendering cells more mutation-prone and potentially promoting tumor initiation. Building on this, the present study characterized the types and frequencies of IGFBP1 mutations in esophageal cancer (ESCA). Among TCGA-ESCA patients, 4.89% harbored IGFBP1 genetic alterations, with amplification being the predominant mutation type (4.35%). Notably, IGFBP1 mutations were associated with shorter overall survival (OS) in ESCA patients. Based on relevant pathway studies, we speculate that IGFBP1 genetic alterations may lead to abnormal activation of downstream pathways and disturb the homeostasis of the IGF signaling axis, which could potentially accelerate ESCA progression. This inference needs experimental verification. Gene amplification typically results in increased copy number and protein overexpression, positioning amplified genes as potential targets for targeted therapies—such as inhibitors or monoclonal antibodies. These mutations can also serve as prognostic markers, aiding in the identification of high-risk patients and informing more aggressive treatment strategies.

GO analysis indicated that IGFBP1 was significantly enriched in metabolic regulation, cell polarity maintenance, immune response, and other pathways, suggesting its multifunctional biological role. Metal ions (such as Fe^2+^, Cu^2+^, Zn^2+^, Ca^2+^, etc.) serve as key regulatory factors in cell proliferation, oxidative stress, and signal transduction, and their abnormal transport can promote tumor development. The enrichment in organic anion transport implies that IGFBP1 may be involved in drug metabolism or chemotherapy resistance, which might be correlated with poor clinical prognosis. IGFBP1 may contribute to tumor invasion and resistance (e.g., in liver and breast cancer) by regulating ion transport and steroid metabolism. The enrichment of apical polarity-related genes could reflect their roles in tissue barriers (e.g., skin and intestines) or epithelial tumors.

KEGG enrichment analysis indicated that neuroactive ligand–receptor interactions, the calcium signaling pathway, and the cAMP signaling pathway might be involved in tumorigenesis mediated by IGFBP1. Studies have demonstrated that certain tumors (e.g., breast cancer and neuroendocrine tumors) exploit the paracrine or autocrine effects of neurotransmitters (such as epinephrine and serotonin) to establish a pro-tumor microenvironment. It is therefore presumed that IGFBP1 may indirectly modulate these interactions by regulating IGF bioavailability. IGF-1 receptor (IGF-1R) activation triggers the release of intracellular calcium ions (Ca^2+^), while IGFBP1 regulates the activity of IGF-1 by binding to it, which may further affect calcium signaling transduction. Abnormal calcium signaling is closely related to tumor cell proliferation, apoptosis resistance, and metastasis [40]. The Ca^2+^ concentration in tumor cells is many times higher than that in normal cells [41]. This elevated Ca^2+^ may be attributed to increased expression of Ca^2+^ channels on the plasma membrane and depletion of intracellular Ca^2+^ stores, which together promote Ca2+-dependent proliferation pathways and inactivation-induced death pathways. Calcium oscillations activate transcription factors (e.g., NFAT and CREB [42]), driving oncogene expression. Calcium-dependent enzymes (such as calmodulin kinase, CaMK) promote epithelial–mesenchymal transition (EMT), enhancing tumor invasiveness, metastatic potential, and treatment resistance [43].

The cAMP pathway has dual properties (oncogenic or antioncogenic), depending on the type of tumor. For example, in melanoma [44], cAMP promotes proliferation; in colon cancer [45], high cAMP may inhibit growth; and in esophageal cancer, in vitro experiments [46] show that cAMP analogues (such as 8-Br-cAMP) can inhibit the proliferation of esophageal squamous cell carcinoma cells. Combining enrichment results and published data, IGFBP1 may participate in the regulation of the above pathways and serve as a potential biomarker for evaluating tumor progression and treatment response. The exact mechanisms require further experimental exploration.

Chinese scholar Lin Yiwei reported that the epithelial–mesenchymal transition (EMT) was inhibited in cells with high IGFBP1 expression. It was found that the ERK signaling pathway was markedly activated in IGFBP1-overexpressing cells, whereas it was significantly suppressed in cells with low IGFBP1 expression. We speculate that IGFBP1 may indirectly regulate cell migration and invasion by modulating the ERK1/2 signaling pathway. A positive feedback loop may exist between IGFBP1 and the ERK1/2 pathway to jointly regulate EMT, cell migration and invasion, but this hypothesis remains to be validated [47].

In summary, IGFBP1 is highly expressed in ESCA and shows potential as a diagnostic biomarker for early-stage esophageal cancer. Additionally, IGFBP1 gene mutations in ESCA patients are significantly associated with overall survival (OS). IGFBP1 expression correlates with immune cell infiltration in esophageal tumors and is involved in multiple tumor cell biological processes. This study provides insights into the potential molecular mechanisms of IGFBP1 in esophageal cancer, as well as its interactions with related immune cells and the tumor microenvironment, thus contributing to further research in this field. Due to the small sample size of this study, the exact molecular mechanisms influencing the prognosis of ESCA patients remain to be further elucidated, and the conclusions have certain limitations. Therefore, subsequent studies should supplement cellular and animal experiments to validate their specific mechanisms underlying esophageal cancer cell proliferation and invasion, design large-scale cohort studies to validate their accuracy in diagnosing early-stage esophageal cancer and their clinical application value, and promote the precision and individualization of tumor treatment.

In addition, this study lacks experimental validation of the IGF, PI3K/AKT, and MAPK signaling pathways. The potential crosstalk between these signaling cascades and the EMT process was analyzed merely based on previously published evidence, without supportive experimental data from our present study.

The number of normal tissue samples in the TCGA cohort is relatively small. Such an imbalance in sample size between tumor and normal groups may weaken the statistical power of differential expression analysis. It increases the risk of false positive or false negative results, and the expression patterns of screened genes may fail to fully reflect the actual characteristics of normal tissues. In addition, a small sample size reduces the stability and generalizability of analytical conclusions, and the findings may not be well extrapolated to broader patient populations.

## Figures and Tables

**Table 1:** RT-qPCR primer sequences.

Gene	Forward Primer (5′-3′)	Reverse Primer (5′-3′)
IGFBP1	GGCTCTCCATGTACCAACA	CCATTCCAAGGGTAGACGCA
β-actin	CAACTGGGACGACATGGAGAAA	GATAGCAACGTACATGGCTGGG

**Table 2:** siRNA sequences.

siRNAs	Sequence (5′-3′)
siIGFBP1	GGAGAMGAGAUCUACAAAU
siCtrl	UUCUCCGAACGUGUUCACGU

## Data Availability

The original data presented in this study are openly available in [GEO] at [GSE45670 (accessed on 2 September 2025)], [TCGA] at [UCSC Xena (accessed on 1 September 2025)], [GEPIA] at [http://gepia.cancer-pku.cn/detail.php?gene=IGFBP1, (accessed on 19 September 2025)], [GEPIA2] at [http://gepia2.cancer-pku.cn/#correlation, (accessed on 25 September 2025)], and [timer2] at [http://compbio.cn/timer2/, (accessed on 17 September 2025)].
